# PPARγ Ligands Decrease Hydrostatic Pressure-Induced Platelet Aggregation and Proinflammatory Activity

**DOI:** 10.1371/journal.pone.0089654

**Published:** 2014-02-28

**Authors:** Fang Rao, Ren-Qiang Yang, Xiao-Shu Chen, Jin-Song Xu, Hui-Min Fu, Hai Su, Ling Wang

**Affiliations:** 1 Department of Cardiology, Guangdong General Hospital, Guangzhou, PR China; 2 Guangdong Academy of Medical Sciences, Guangzhou, PR China; 3 Department of Cardiology, The second affiliated hospital of Nanchang University, Nanchang, PR China; 4 Jiangxi Province Blood center, Nanchang, PR China; University of Illinois College of Medicine, United States of America

## Abstract

Hypertension is known to be associated with platelet overactivity, but the direct effects of hydrostatic pressure on platelet function remain unclear. The present study sought to investigate whether elevated hydrostatic pressure is responsible for platelet activation and to address the potential role of peroxisome proliferator-activated receptor-γ (PPARγ). We observed that hypertensive patients had significantly higher platelet volume and rate of ADP-induced platelets aggregation compared to the controls. In vitro, Primary human platelets were cultured under standard (0 mmHg) or increased (120, 180, 240 mmHg) hydrostatic pressure for 18 h. Exposure to elevated pressure was associated with morphological changes in platelets. Platelet aggregation and PAC-1 (the active confirmation of GPIIb/IIIa) binding were increased, CD40L was translocated from cytoplasm to the surface of platelet and soluble CD40L (sCD40L) was released into the medium in response to elevated hydrostatic pressure (180 and 240 mmHg). The PPARγ activity was up-regulated as the pressure was increased from 120 mmHg to 180 mmHg. Pressure-induced platelet aggregation, PAC-1 binding, and translocation and release of CD40L were all attenuated by the PPARγ agonist Thiazolidinediones (TZDs). These results demonstrate that platelet activation and aggregation are increased by exposure to elevated pressure and that PPARγ may modulate platelet activation induced by high hydrostatic pressure.

## Introduction

The relationship between hypertension and platelet function is a subject of increasing interest. Recent research have revealed that both platelet clumping and cohesion are sthenic in patients with hypertension [1], [2], and indices of platelet function including platelet size, aggregation and the release of platelet activation markers are all increased in subjects with hypertension [3]. Platelet activation is accompanied by a marked increase in the membrane levels of glycoproteins (GP) IIb/IIIa, and receptor–ligand interactions between these molecules contribute to platelet aggregation [4], [5]. PAC-1 (antibody that recognizes conformational change of the GPIIb/IIIa complex) binding and P-selectin are the two main active marker of activated platelets. The activation and aggregation of platelets in patients with hypertension can predispose to serious thrombotic disease and aggravate the development of ischemic heart and cerebrovascular disease.

As a new immune cell, platelets not only play a central role in hemostasis but also are key regulators of inflammation and immunity. The role of platelets in inflammation is thought to be mediated through CD40 ligand (CD40L)–CD40 interactions. The co-stimulatory molecule CD40 and its ligand CD40L (CD154, GP139) are expressed on not only immune cells, including B cells, T cells, and monocytes, but also on non-immune cells, including platelets, endothelial cells, and fibroblasts et al [6]–[9]. Besides a membrane-bound form, CD40L also exists as a soluble molecule; soluble CD40L (sCD40L), mainly derives from activated platelets and T cells after stimulation [10], [11]. Ensuing cleavage and release of CD40L from the surface has been reported to be associated with increased cardiovascular risk in a healthy population [12], and CD40-CD40L contributes to the pathogenesis of atherosclerotic, thrombotic, and inflammatory conditions [13]–[15]. However, the role of blood pressure in platelet aggregation, immunoregulation and inflammation remains unclear, despite abundant evidence that increased blood pressure can lead to functional impairment of platelets, and the intracellular pathways involved remain obscure.

The peroxisome proliferator-activated receptor-γ (PPARγ), a member of the nuclear receptor superfamily of ligand-dependent transcription factors, is involved in the regulation of inflammation [16]. In activated macrophages and vascular smooth muscle cells, agonists of PPARγ inhibit the expression of many pro-inflammatory genes [17], [18]. PPARγ is highly expressed in platelets, and PPARγ activation can inhibit platelet activation and decrease CD40L release [19], [20]. Nevertheless, the role of PPARγ in countering the adverse effects of hypertension on platelet function has not been studied.

Although clinical findings have pointed to a close link between platelet function and hypertension, it is possible that other factors in addition to blood pressure also contribute, including obesity, diabetes, and hyperlipemia. To exclude these factors we sought to address the effects of blood pressure on platelet activation in an in vitro system in which hydrostatic pressure can be varied systematically. We report on changes in platelet morphology and function in response to increased hydrostatic pressure and on experiments that address the potential role PPARγ in platelet protection.

## Materials and Methods

### Ethics Statement

This investigation was in accordance with the principles outlined in the Declaration of Helsinki and was approved by the Research Ethics Committee, the second affiliated hospital of Nanchang University (NO. 2011015). All patients and volunteers provided written informed consent.

### Patients

Two hundred and four patients diagnosed as essential hypertension were recruited, 65 patients (group 1; 53±3.9 years, Male:40, Female:25) with grade 1 (mild; SBP levels 140–159 mmHg, DBP levels 90–99 mmHg) hypertension, 77 patients (group 2; 58±4.2 years, Male:47, Female:30) with grade 2 (moderate; SBP levels 160–179 mmHg, DBP levels 100–109 mmHg) hypertension, 62 patients (group 3; 60±7.4 years, Male:38, Female:24) with grade 3 (severe; SBP levels ≥180 mmHg, DBP levels ≥110 mmHg) hypertension were included in the study. Exclusion criteria were women of child bearing potential in whom pregnancy could not be ruled out, secondary hypertension, coexisting medical conditions such as diabetes, coronary artery disease, peripheral vascular disease, previous stroke or myocardial infarction, previous malignant hypertension or congestive cardiac failure, and infectious diseases.

In addition, 89 health subjects (49±7.3 years, Male:51, Female:37) with normal blood pressure (<140/90 mmHg) as measured during routine physical examination were recruited as the control group.

All blood samples were drawn in K2EDTA-anticoagulated blood and the platelet parameters were measured by the Sysmex SE-2100 (Sysmex Corporation, Kobe, Japan) fully automated haematology analyzer. The specimens were analyzed within 1 hour from venesection.

### Hydrostatic pressure device

To manipulate hydrostatic pressure in cell culture in vitro a device was designed (Patent No. ZL 2006 2 0097266.3, China) comprising a sealed container and electric pump. The device operates by injecting compressed gas (typically 5% CO_2_ in air) into the sealed container; an outlet valve is used to regulate the rate of gas loss and internal pressure. To mimic rhythmic pressure oscillations that accompany the heart beat in vivo, the pump can be adjusted to inject gas in a pulsatile manner (in the range 0–100/min). In the experiments described here a pulse rate of 60/min was employed and the difference between minimum and maximum pressures was maintained at 20 mmHg. The pressure device containing cell culture plates was placed into a standard incubator to maintain constant temperature.

### Platelet collection and culture

Apheresis platelets were collected from healthy volunteers (aged 20–30 years) with informed consent. Blood separation was performed using a CS-3000 Plus separator (Baxter International) and platelets were collected in a CPDA-1 blood collection bag (Baxter Healthcare, Deerfield, IL). Platelet counts were typically 300×10^6^/m1. Aliquots (2 ml) of platelet-rich plasma (PRP) were exposed to 0, 120, 180, or 240 mmHg at 22°C and pressure oscillated at 60/min. Aliquots were removed for analysis of platelet aggregation and morphology. For transmission electron microscopy, flow cytometry, western blotting and PPARγ activity determinations, samples were centrifuged (2919 g, 10 min) and washed with Tyrode's (HT) buffer (mM) (NaCl 137, KCl 2.8, MgCl_2_ 1, NaHCO_3_ 12, Na_2_HPO_4_ 0.4, glucose 5.5, HEPES 10, pH 7.4).

### Transmission electron microscopy

Washed platelets were prepared and fixed in Karnovsky fixative. Samples were then post-fixed in 1% (w/v) osmium tetroxide buffer, washed with distilled water, dehydrated through increasing concentrations of ethanol, replaced with propylene oxide and acetone, and embedded in Eponate 812 (Ted Pella Inc., USA). Ultrathin sections (50–60 nm) were cut using a Power-Tome PC Ultracut (RMC Company, USA) and sections were counterstained with ethanolic uranyl acetate and lead nitrate. Sections were observed and photographed on a JEM-1230 transmission electron microscope (JEOL, Japan) at 80 kV.

### Platelet morphology

Platelet parameters, including platelet count (PLT), mean platelet volume (MPV), platelet distribution width (PDW) and platelet-large cell ratio (P-LCR) were determined using an automated cell counting device (Sysmex XE-2100, Japan).

### Platelet aggregation

ADP (20 µM, Sigma-Aldrich, USA) was used as the stimulus for platelet aggregation. All aggregation studies were performed in duplicate using a four-channel aggregometer (PACKS-4; Helena Laboratories, Beaumont, TX, USA).

### Western blot analysis

Platelet proteins were extracted by a standard technique and aliquots (30 µg) were diluted with 4×Loading buffer (Invitrogen, USA), heated at 95°C for 5 min, and fractionated by 10% (for PPARγ, CD40L and GAPDH) polyacrylamide gel electrophoresis in the presence of SDS. Gels were transferred to nitrocellulose membranes (Amersham, USA), blocked with dried skimmed milk powder in Tris-buffered saline Tween (TBST) for 2 h at room temperature before overnight incubation at 4°C with the primary antibodies. These were rabbit polyclonal antibodies to PPARγ and CD40L (1∶500; Santa Cruz Biotechnology, CA); anti-GAPDH (1∶1000; Cell Signaling Technology, USA) was used as the internal control. After washing in TBST, the membranes were incubated for 1 h with horseradish peroxidase (HRP)-conjugated anti-rabbit IgG (KPL, USA) in blocking solution. Protein bands were visualized using electrochemiluminescence (ECL) reagents (Pierce, USA), and films were evaluated densitometrically using Gelpro analyzer 4.0 software (Media Cybernetics, USA).

### PPARγ activity assay

Concentrated platelets were washed twice and lysed with hypotonic buffer (10 mM HEPES-KOH pH 7.9, 1.5 mM MgCl_2_, 10 mM KCl, 0.5 mM dithiothreitol, 0.5% Nonidet P-40, 0.2 mM phenylmethylsulfonyl fluoride); 10 µg aliquots of platelet extracts were incubated in each well of the TransAM PPARγ assay kit (Active Motif, CA) and PPARγ DNA binding was determined according to the manufacturer's protocol.

### Flow cytometry of PAC-1 and CD40L

Platelet samples were centrifuged and washed with modified HT buffer, incubated with 10 µl anti-CD40L (Becton Dickinson, USA) or 10 µl PAC-1-FITC (Becton Dickinson, USA) or FITC-labeled IgG1 isotype control (Becton Dickinson, USA) or RGDS+PAC-1^FITC^ (10 µl, 5 mg/ml RGDS)control (Becton Dickinson, USA) for 30 min in the dark at room temperature. Samples were diluted with 800 µl of HT buffer and analyzed on a Becton Dickinson FACSCalibur flow cytometer (Becton Dickinson, USA). FITC-labeled IgG1 isotype and RGDS+PAC-1^FITC^ control were used to assess nonspecific binding. Data acquisition and analysis were performed using CELLQuest software (Becton Dickinson, USA) and results were expressed as the mean fluorescence index (MFI) of CD40L or PAC-1.

### Enzyme-linked immunosorbent assay (ELISA) of soluble CD40L

Levels of soluble CD40L (sCD40L) in the medium were measured by ELISA kit (R&D Systems, USA) according to the manufacturer's protocol.

### Data analysis

Results are expressed as means ± SEM. Statistical significance was evaluated with the use of one-way ANOVA followed by a Newman–Keuls test to compare individual means. Differences were considered significant at *P*<0.05.

## Results

### The parameters of platelet volume and platelet aggregation in patients with various grades of hypertension

**Figure 1:**
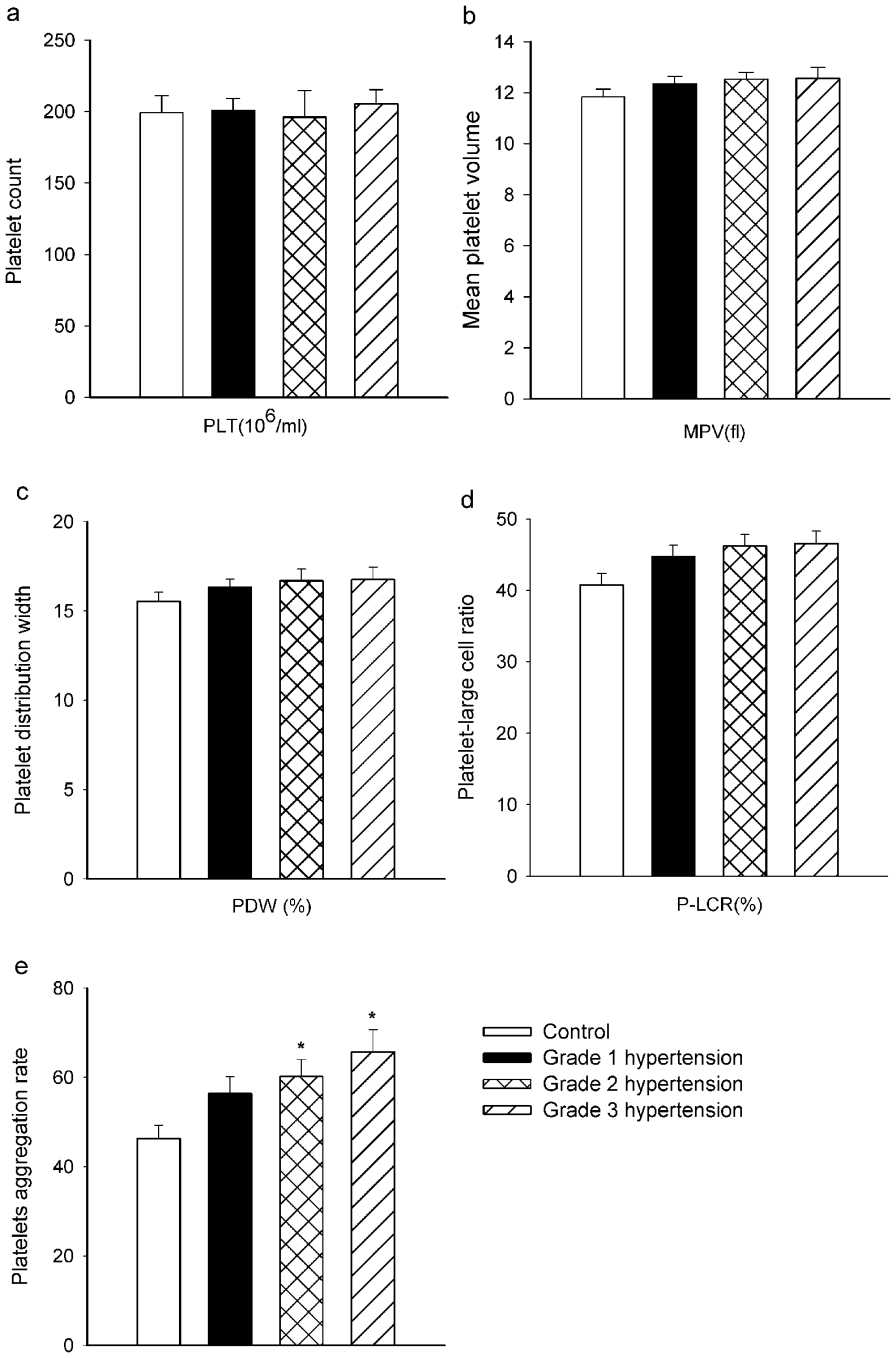
Platelet parameters and the rate of platelet aggregation in patients with various hypertension and controls. Platelet parameters, including platelet count (PLT), mean platelet volume (MPV), platelet distribution width (PDW) and platelet-large cell ratio (P-LCR) ADP (20 µM) was used as the stimulus for platelet aggregation. *P<0.05 versus control; **P<0.01 versus control.

We studied 204 previously diagnosed patients with various grades of hypertension, and compared them with 89 normotensive controls who were matched for age and sex. We found raised platelet volume, increased platelet PDW and P-LCR among the hypertensive patients (*P*<0.05). The difference in mean platelet count was not statistically different. The rate of ADP (20 µM)-induced aggregation of platelets was higher (*P*<0.05) in patients with grade 2 hypertension (60.2±3.8%), grade 3 hypertension (65.7±4.9%) as compared to healthy subjects (46.3±2.9%) (Figure 1).

### Elevated hydrostatic pressure causes morphological and functional changes in platelets

**Figure 2:**
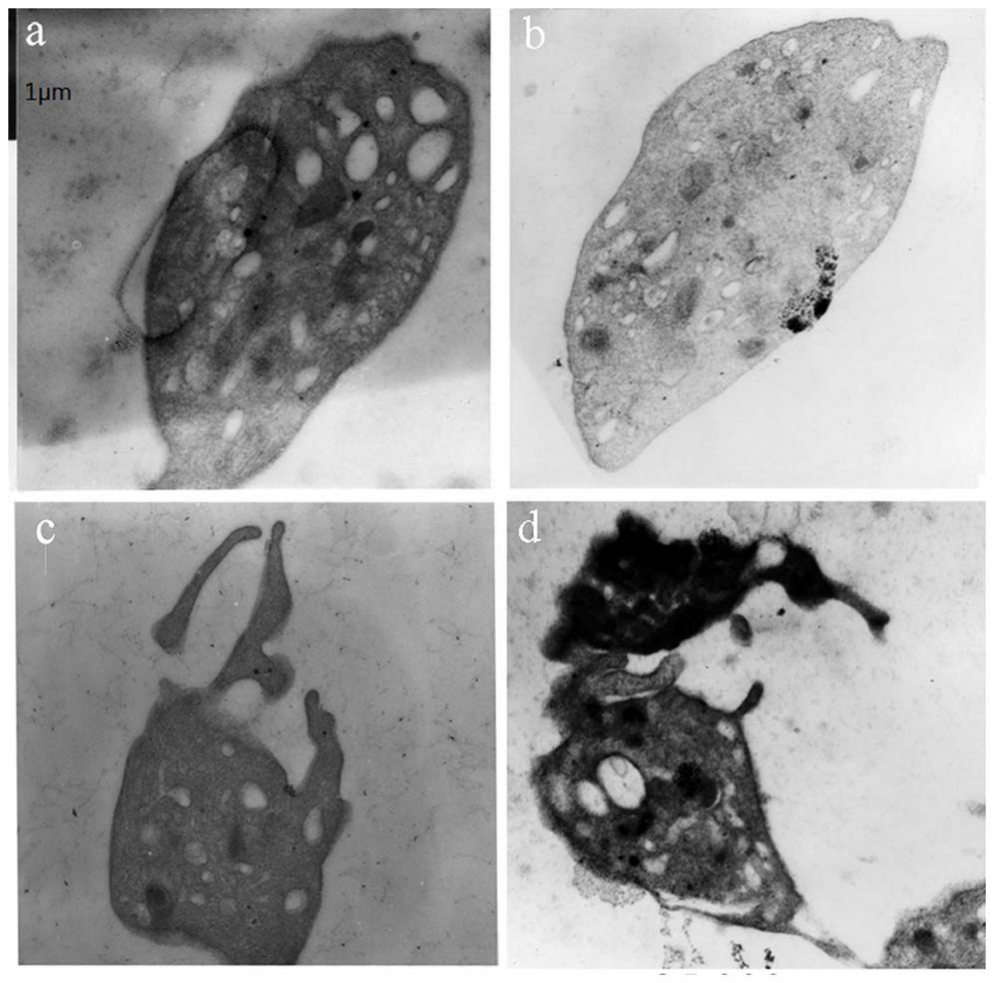
Transmission electron microscopy (TEM) of platelets exposure to (a, b) 0 mmHg and (c, d) 180 mmHg. High pressure-treated platelets showed prominent pseudopodial protrusion of cytoplasm and the absence of dense granules. Scale bar represents 1 µm.

Primary human platelets were cultured at 22°C for 18 h at elevated hydrostatic pressure mimicking moderate to severe hypertension (180 mmHg). As shown in Figure 2, under these conditions platelet size morphology was markedly diverse, with prominent pseudopodial protrusion of cytoplasm and the absence of dense granules. A small number of collapsed cells were also observed. Quantitation of morphological changes revealed that the per platelet ratio of abnormal morphologies increased from 12.5% in control samples to 71.5% in platelets exposed to high pressure (*P*<0.001).

**Figure 3:**
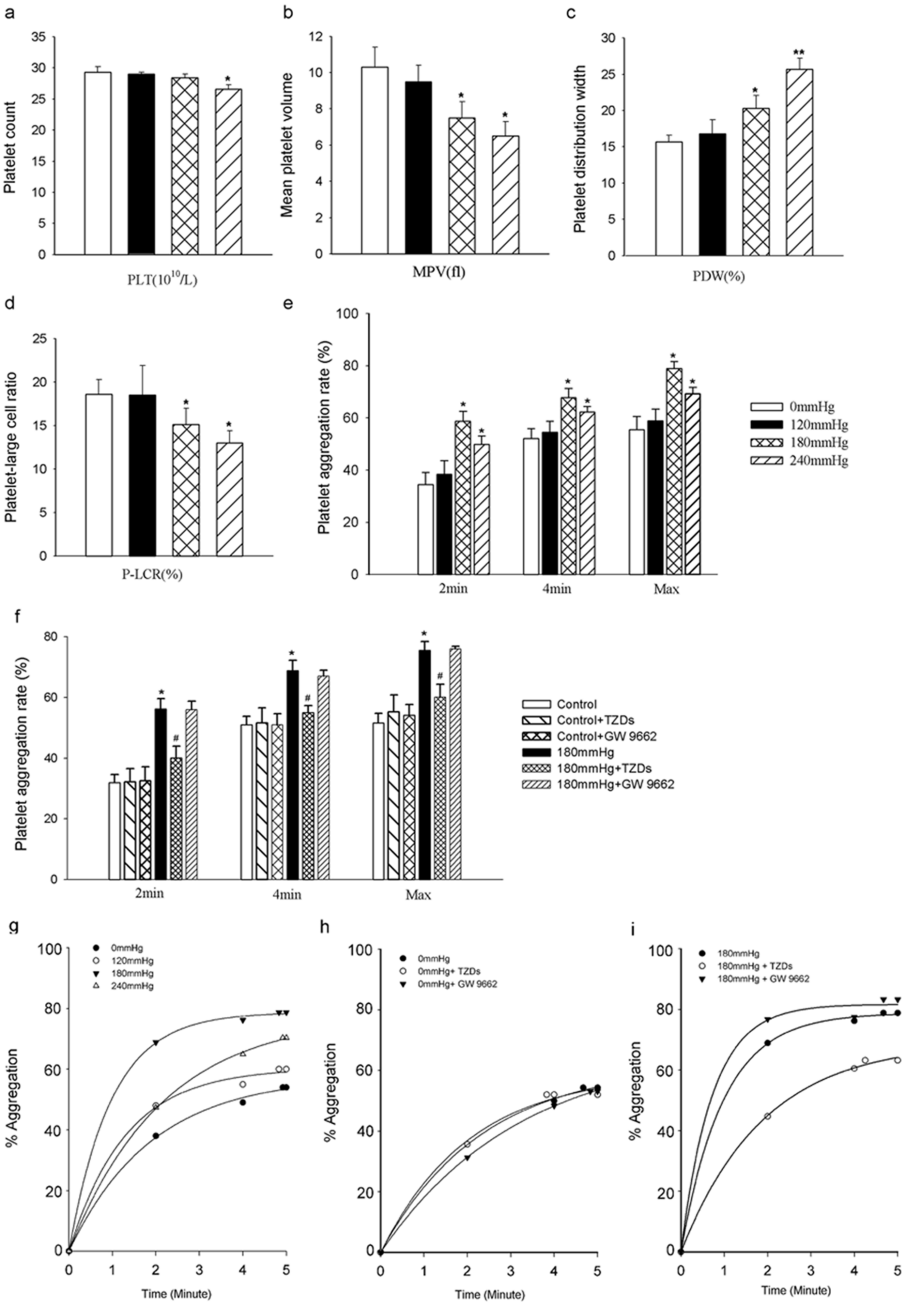
Platelet morphology parameters and platelet aggregation rate after exposure to different hydrostatic pressures for 18 h in vitro. *a–d*, Bar graphs showing means ± SEM of PLT, MPV, PDW and P-LCR at 18 h in pressure-treated platelets compared to controls. *e*, ADP (20 µM) was used to induce platelet aggregation cultured under different pressures. The extent of aggregation was determined by the area under the aggregation curve from zero to five minutes following ADP exposure. *f*, Platelet aggregation rate (%) in control or at 180 mmHg in the absence or presence of the PPARγ agonist TZDs or the PPARγ antagonist GW9662. Mean values for each group were determined from four separate experiments each performed in duplicate. *P<0.05 versus control; ^#^ *P*<0.05 versus 180 mmHg. *g–i*: The typical aggregation traces of platelet aggregation rate under different pressures, or at 0 mmHg and 180 mmHg in the absence or presence of TZDs or GW9662.

We then examined several specific parameters relating to volume (MPV), width (PDW) and the platelet-large cell ratio (P-LCR) as a function of hydrostatic pressure. Compared to control (0 mmHg), both MPV and P-LCR were reduced while PDW increased in the 180 and 240 mmHg groups; there were no differences in MPV, P-LCR and PDW in platelets cultured at 120 mmHg (Figure 3a–d). Culture at 120 and 180 mmHg had no effect on PLT but a significant decrease in PLT was observed (29.3±0.9 to 26.6±0.7; n = 8, *P*<0.05), when the pressure was increased to 240 mmHg (Figure 3a). Importantly, high hydrostatic pressure (180 and 240 mmHg) caused a marked increase in platelet activation (Figure 3e). We concluded that whereas incubation at normal physiological pressure (∼120 mmHg) was without effect on platelet morphology and function, incubated at high pressure (180 and 240 mmHg) produced marked changes in platelet morphology and aggregation.

### PPARγ activation inhibits platelet aggregation induced by elevated hydrostatic pressure

To investigate the potential role of PPARγ in platelet aggregation induced by 180 mmHg, platelet aggregation parameters were measured after exposure to 180 mmHg for 18 h in the presence or absence of a specific PPARγ agonist, Thiazolidinediones (TZDs, Cayman Chemical, Ann Arbor, MI), or a specific PPARγ antagonist, Gw9662 (Cayman Chemical, Ann Arbor, MI). As shown in Figure 3f, the increase in platelet aggregation induced by exposure to 180 mmHg was significantly reduced by TZDs (0.6 µM), whereas Gw9662 (0.1 µM) was without effect. This finding argues that PPARγ activation can inhibit platelet aggregation induced by elevated hydrostatic pressure.

### PPARγ activity is up-regulated by increased hydrostatic pressure

**Figure 4:**
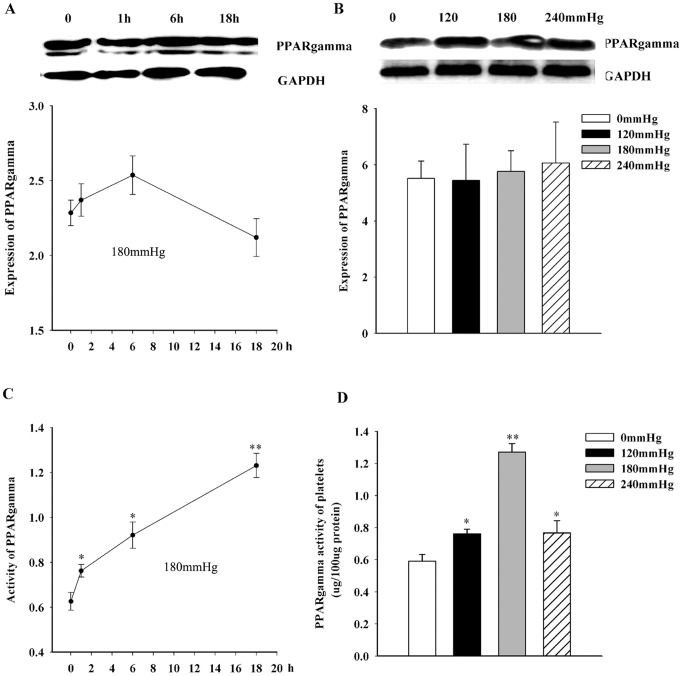
Expression and activity of PPARγ in control and pressure-treated platelets. *A,B*, *Top*: representative western blot analysis of PPARγ protein at different timepoints in platelets exposed to 180 mmHg, and different pressures for 18 h. GAPDH provided the internal control. Each blot represents one of three experiments. *Bottom*: densitometric analysis of PPARγ protein in different timepoints in 180 mmHg, or control and pressure-treated platelets for 18 h. *C–D*, PPARγ activity was determined using a commercial assay kit at different timepoints under 180 mmHg conditions, or in platelets exposed to different pressures for 18 h. *P<0.05 versus control; **P<0.01 versus control.

To investigate the potential involvement of changes in PPARγ expression and activity in modulating platelet activation induced by hydrostatic pressure, western blotting was used to measure levels of PPARγ protein in platelets exposed to 180 mmHg for 18 h. As shown in Figure 4A,B, elevated hydrostatic pressure had no effect on the levels of PPARγ protein. By contrast, the activity of PPARγ was significantly up-regulated in platelets exposed to elevated pressure, increasing from 0.6±0.04 to 0.8±0.03 at 120 mmHg (n = 6, *P*<0.05) and to 1.3±0.05 at 180 mmHg (n = 6, *P*<0.01), whereas no further increase was observed following exposure to 240 mmHg (0.8±0.08, n = 6) (Figure 4D). The increase of PPARγ activity was time-dependent (Figure 4C).

### Exposure to elevated hydrostatic pressure up-regulates markers of platelet activation

**Figure 5:**
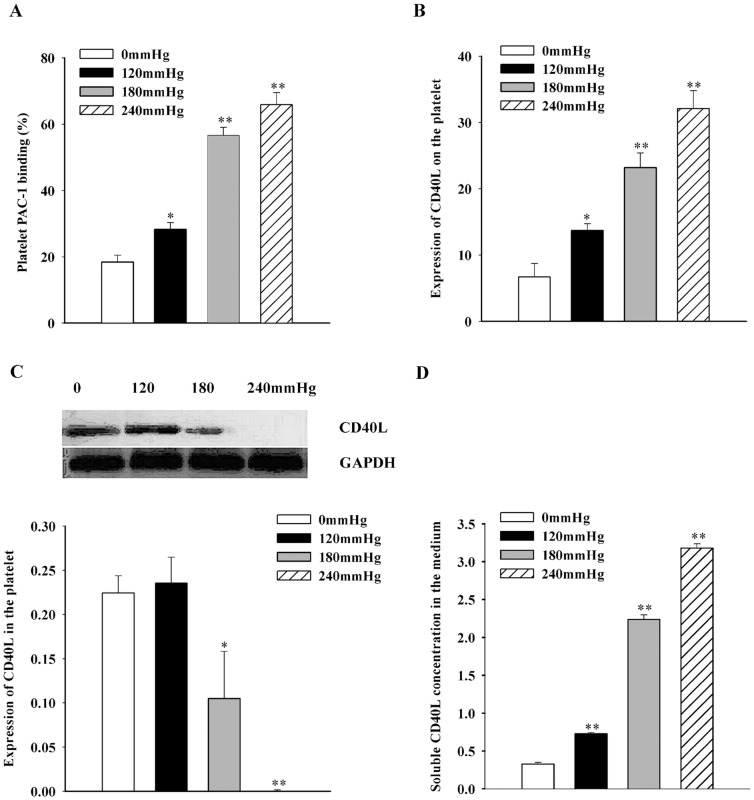
PAC-1 binding and CD40L expression in platelets exposed to different pressures for 18 h. *A–B*, Analysis of platelet surface PAC-1 binding and CD40L expression levels by flow cytometry after treated with different pressures. Mean values for each group were determined from three separate experiments each performed in duplicate. *C*, *Top*: representative western blot analysis of CD40L expression in pressure-treated platelets; GAPDH provided the internal control. *Bottom*: densitometric analysis of CD40L expression in lysates of pressure-treated platelets and controls. *D*, Levels of soluble CD40L in the culture medium determined by ELISA after treated with different pressures. *P<0.05 versus control; **P<0.01 versus control.

To determine whether elevated hydrostatic pressure induced platelet activation, flow cytometry was used to determine the expression levels of two activation markers, PAC-1 binding and CD40L. As shown in Figure 5A, elevated hydrostatic pressure produced a significant increase in the levels of PAC-1 binding, from 18.4±2.1 in controls to 28.3±2.0 in platelets exposed to 120 mmHg (n = 6, *P*<0.05), to 56.6±2.5 at 180 mmHg (n = 6, *P*<0.01), further increasing to 65.9±3.6 at 240 mmHg (n = 6, *P*<0.01). The membrane levels of CD40L increased from 6.7±2.0 in controls to 13.7±1.0 (n = 6, *P*<0.05), 23.2±2.2 (n = 6, *P*<0.01), and 32.1±2.7 (n = 6, *P*<0.01) in platelets exposed to 120, 180, or 240 mmHg, respectively (Figure 5B). We also measured protein levels of CD40L in platelet lysates, and found that CD40L protein levels decreased from 0.2±0.02 to 0.1±0.05 (n = 6, *P*<0.05) in platelets exposed to 180 mmHg, and to 0.0007±0.0006 (n = 6, *P*<0.01) at 240 mmHg (Figure 5C). Conversely, levels of soluble CD40L in the culture medium increased from 0.3±0.02 in control samples to 0.7±0.01 at 120 mmHg, 2.2±0.06 at 180 mmHg; and 3.2±0.06 at 240 mmHg (n = 6, *P*<0.01) (Figure 5D). This finding indicates that exposure to elevated pressure leads to translocation of CD40L to the membrane and release into the medium.

### Platelet activation and CD40L release induced by elevated hydrostatic pressure can be blocked by PPARγ activation

**Figure 6:**
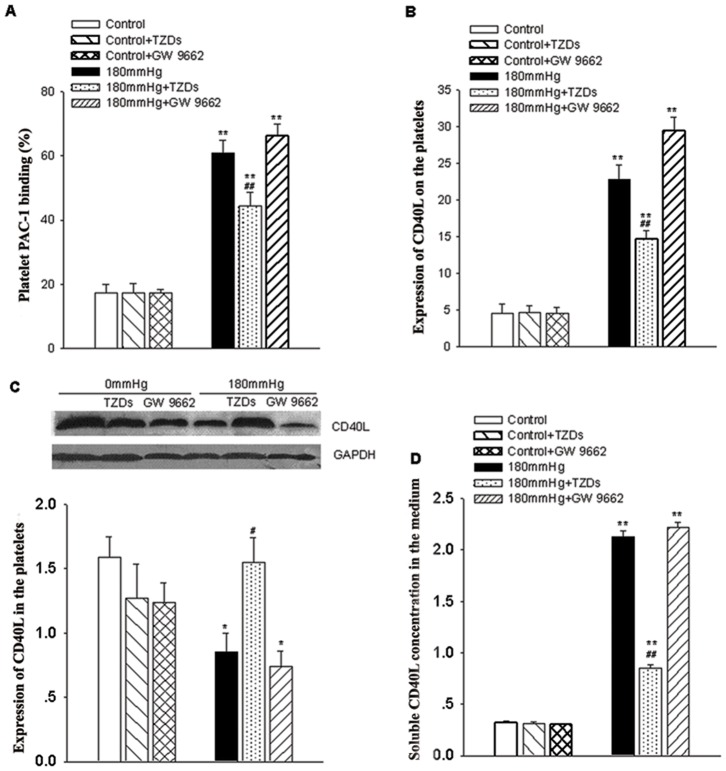
Expression of platelet activation markers PAC-1 binding and CD40L expression in platelets exposed to high hydrostatic pressure, role of PPARγ. Platelets were exposed to control (0 mmHg) or 180 mmHg for 18 h in the presence or absence of the PPARγ ligands TZDs or Gw9662. *A–B*, Levels of PAC-1 binding and CD40L expression analyzed using flow cytometry. *C*, Representative western blot and densitometric analysis of CD40L expression in lysates of pressure-treated platelets and controls in the presence or absence of the PPARγ ligands. GAPDH was the internal control. *D*, Soluble CD40L in the culture medium was measured by ELISA after treated with different pressure and PPARγ ligands. *P<0.05, **P<0.01, versus control; ^#^ *P*<0.05, ^##^ *P*<0.01, versus 180 mmHg.

To study whether changes in PPARγ activity modulated the increase in PAC-1 binding levels induced by elevated pressure, platelets were exposed for 18 h to 0 or 180 mmHg in the presence or absence of TZDs, or GW9662; PAC-1 binding percentages were found to be significantly lowered in platelets cultured in the presence of TZDs (60.7±4.1 in 180 mmHg group versus 44.4±4.2 in 180 mmHg+TZDs; n = 6, *P*<0.05), whereas GW9662 was without effect on PAC-1 binding (Figure 6A).

We also examined levels of CD40L expression detected by flow cytometry. As before, exposure to elevated pressure caused a marked increase in membrane CD40L levels (from 4.5±1.24 to 22.8±1.99; n = 6, *P*<0.01) and a marked decrease in the cytoplasm of platelet (from 1.59±0.16 to 0.85±0.15; n = 6, *P*<0.01), but the adverse changes were significantly attenuated by the PPARγ agonist-TZDs (from 22.81±1.99 to 14.6±1.12, n = 6, *P*<0.01; from 0.85±0.15 to 1.55±0.19; n = 6, *P*<0.01) (Figure 6 B,C). And TZDs also reduced the release of soluble CD40L into the culture medium, whereas Gw9662 had no effect on the translocation or release of CD40L (Figure 6D). This finding indicates that activation of PPARγ attenuates platelet activation induced by elevated hydrostatic pressure.

## Discussion

We have demonstrated that hypertensive patients had significantly higher platelet volume and rate of platelets aggregation compared to the controls. And we have also found that culture under conditions of elevated hydrostatic pressure (180 mmHg or 240 mmHg) simulating moderate to severe hypertension leads to marked changes in platelet morphology and activation. High pressure led to increased platelet aggregation and up-regulation of PAC-1 binding levels, a marker of platelet activation. Elevated pressure also led to CD40L translocation to the membrane and release into the culture medium. At the same time, exposure to elevated pressure was accompanied by up-regulation of the activity of PPARγ, and a specific PPARγ agonist was able to prevent platelet activation, suggesting that the up-regulation of PPARγ under high pressure conditions is likely to attenuate the adverse effects of elevated hydrostatic pressure.

It was previously demonstrated that indices of platelet function including platelet size (measured as MPV) and titre (PLT), and platelet aggregation were increased in subjects with elevated blood pressure [21]. In the present study, we also found that platelet MPV, PDW and P-LCR were increased in patients with hypertension in a pressure-dependent manner. The rate of platelet aggregation was higher in grade 2 and 3 hypertension, compared to patient with grade 1 hypertension and the controls. By contrast, in vitro, both MPV and PLT were reduced by exposure to high pressure (180 mmHg or 240 mmHg). However, it seems likely that the discrepancy can be explained by increased turnover in vivo. Overactivation of platelets and aggregation in hypertension would lead to a faster rate of removal from the circulation, and this is likely to lead to a compensatory up-regulation of platelet production by bone marrow megakaryocytes, thereby increasing platelet titres in patients with hypertension.

Hypertension is associated with an increased risk of thrombotic, as opposed to hemorrhagic, complications such as stroke and myocardial infarction. This could be due, at least in part, to the prothrombotic state associated with inappropriate platelet activation, such as increased platelet aggregation and the release of platelet activation markers [22], [23]. GPIIb/IIIa, a member of the integrin family of cell adhesion molecules, is the most abundant membrane protein of platelets. PAC-1, the active confirmation of GPIIb/IIIa, is thought to reflect platelet activation and adhesion status. In the present study we found that PAC-1 binding levels were significantly increased under high-pressure culture conditions, indicating that increased levels of GPIIb/IIIa were present on the membrane, consistent with platelet activation under elevated pressure.

Recent studies have suggested that platelets also participate in inflammatory reactions. CD40L has been detected in platelets where, after stimulation, it is translocated to the platelet surface [10]. The surface-expressed CD40L is then cleaved from the platelet to generate a soluble fragment (sCD40L) [24], and this process is increased in fresh thrombus platelets [10], and in cardiovascular disease [25], [26]. Although CD40L has been characterized as a marker of thrombotic and inflammatory disease, much less is known about its role in the adverse platelet changes induced by high hydrostatic pressure. In the present study we report that elevated pressure leads to decreased membrane levels of CD40L but increased levels in the medium, indicative of platelet activation. This suggests that CD40L-dependent inflammatory and immunomodulatory processes are likely to be affected in patients with hypertension.

We have also addressed the potential role of PPARγ in platelet responses to elevated hydrostatic pressure. PPARγ is known to be actively involved in the modulation of inflammation and angiogenesis [16], [27], and agonists of PPARγ are reported inhibit the expression of several pro-inflammatory genes [17], [18], [28]. PPARγ is highly expressed in platelets and is involved in the inhibition of platelet aggregation and in the modulation of inflammation [19]. In the present study we found that PPARγ protein levels were not changed by culture at elevated pressure, but the activity of PPARγ was markedly increased at 120 and 180 mmHg, although at 240 mmHg levels fell to those seen at 120 mmHg. The increase in PPARγ activity is likely to serve to alleviate the adverse effects of high pressure, but that the potential protective role of PPARγ is impaired at very high pressure (240 mmHg). We therefore concluded that, at moderately elevated pressure, PPARγ acts to constrain platelet aggregation and inflammatory processes induced by high pressure, but the protective effect is reduced at extremely high pressure.

However, the response to PPARγ activation is puzzling because platelets are anucleate cytoplasts and transcriptional changes are therefore excluded. Nevertheless, nuclear receptors have been implicated in diverse post-transcriptional processes including the induction of signal transduction pathways and mRNA stabilization and localization. Moreover, platelets do contain functional spliceosomes, the complexes responsible for processing pre-mRNAs in the nuclei of other cell types, and signal-dependent splicing is a novel function of platelets that demonstrates remarkable specialization in the regulatory repertoire of this anucleate cell [29]. We surmise that so-far unidentified platelet pressure-receptors are able to transduce the mechanical signal, leading to the activation of intracellular signaling pathways; it is possible that these include the activation of signaling pathways or signal-dependent pre-mRNA splicing, leading to changes in expression levels of specific polypeptides involved in platelet function.

In the present study, PPARγ agonist TZDs was found to inhibit platelet activation through down-regulating the level of PAC-1 binding and the translocation and release of CD40L. Because there is not much DNA in platelets, the increase of PPARγ binding affinity to DNA, which might not play an important role in affecting the activation of platelets. The non-genomic role of PPARγ during platelet activation might inhibit the proinflammatory signaling cascade induced by high hydrostatic pressure. So TZDs might also inhibit platelet aggregation and function by some other off-target effects. As a potent and selective antagonist of full-length PPARγ, GW9662, did not change the activation outcome. This might be due to the activation of PPARγ induced by high pressure might partly counteracted the effect of GW9662. In addition, there might be other pro-inflammatory signal pathways activated by high pressure, which could not be affected by GW9662.

In clinic, TZDs was first used in 1996 to treat diabetes mellitus. They increased glucose and lipid uptake, and improved insulin sensitivity. Although, the effects of TZDs on the risk of cardiovascular events are still controversial [30]. According to our study, we thought that TZDs might be introduced to the patients with hypertension and diabetes, to help reduce the thrombotic events.

## Conclusions

To summarize, we have demonstrated that exposure to elevated hydrostatic pressure in vitro leads to increased platelet activation and aggregation, and that these processes are modulated by PPARγ. The response of platelets to elevated pressure suggests that platelet activation is likely to contribute to the platelet thrombosis and thrombotic disease observed in patients with hypertension.
